# Connectome Gradient‐Based Subtyping of Major Depressive Disorder Reveals Distinct Neurobiological and Transcriptomic Signatures

**DOI:** 10.1155/da/8792696

**Published:** 2026-09-02

**Authors:** Xiaozheng Liu, Zhongwei Guo

**Affiliations:** ^1^ The Second Affiliated Hospital and Yuying Children’s Hospital, Wenzhou Medical University, Wenzhou 325027, Zhejiang, China, wmu.edu.cn; ^2^ Wenzhou Key Laboratory of Structural and Functional Imaging, Wenzhou 325027, Zhejiang, China; ^3^ Tongde Hospital of Zhejiang Province, Hangzhou 310012, Zhejiang, China, zjtongde.com

**Keywords:** functional gradients, functional magnetic resonance imaging, major depressive disorder, subtypes

## Abstract

**Background/Objective:**

Major depressive disorder (MDD) exhibits significant heterogeneity, and identifying these distinct biological subtypes aids clinical intervention. In this study, we employed functional gradient and Hydra clustering methods to distinguish subtypes of depression.

**Method:**

Imaging data were derived from a large‐scale, multicenter MDD imaging database, comprising 1067 patients with MDD and 907 healthy controls (HCs). Based on the principal functional gradient, the Hydra method was employed to distinguish subtypes of MDD. We employed the Mann–Whitney *U*‐test to compare functional gradient abnormalities across different subtypes of MDD while simultaneously conducting imaging transcriptomics analysis.

**Results:**

Using principal functional gradients, we identified two subtypes of MDD. Compared with HCs, subtype 1 exhibited abnormal functional gradient values in the dorsal attention network (DAN), ventral attention network (VAN), limbic network (LIB), frontoparietal network (FTP), and default mode network (DMN); subtype 2 exhibited abnormal functional gradient values in the visual network (VIS), somatosensory network (SMT), DAN, VAN, and DMN. Transcriptomics revealed that subtype 1 functional gradient abnormalities were significantly correlated with theory mind, 5‐hydroxytryptamine (5‐HT) 1B; subtype 2 functional gradient abnormalities were significantly correlated with motor function, dopamine D2 receptor, and norepinephrine transporter (NET).

**Conclusions:**

Our research deepens the understanding of the biological diversity of MDD, providing a biologically grounded framework for stratifying patients, which may inform the future design of hypothesis‐driven clinical trials targeting specific cognitive or sensorimotor circuits.

## 1. Introduction

Major depressive disorder (MDD) is a prevalent mood disorder defined by persistent low mood and a marked loss of interest or pleasure. Its pathophysiology is multifactorial, involving genetic, environmental, and neurobiological determinants, yet remains only partially elucidated [1]. Clinically, patients with MDD display pronounced heterogeneity in symptomatic presentation, illness trajectory, and therapeutic outcomes, which likely reflects interindividual variations in underlying neurobiological disturbances [2, 3]. This diversity engenders a fundamental discordance between observable symptom profiles and their biological substrates—such that identical clinical manifestations may arise from distinct etiological pathways. Consequently, this heterogeneity imposes an imperative for personalized diagnostic and intervention approaches, underscoring the need to devise mechanism‐based precision strategies tailored to individual pathophysiological features [4].

Monoamine deficiency is one of the key factors contributing to the onset of depression [5]. Chronic antidepressant treatment increases 5‐hydroxytryptamine (5‐HT) release by reducing 5‐HT1B receptor expression and efficacy in the dorsal raphe nucleus [6]. Administration of 5‐HT1B receptor antagonists can prevent memory impairment and promote learning [7]. Deep brain stimulation improves depressive and anxiety symptoms while reducing dopamine D2 receptor (DRD2) binding in the amygdala, caudate nucleus, and substantia nigra [8]. Ras homolog enriched in striatum modulates DRD2‐mediated antidepressant‐like effects in ovariectomized mice [9]. D2 receptor activity in the motor cortex circuit influences motor skill acquisition induced by movement [10]. Degeneration of locus coeruleus norepinephrine neurons is considered the basis for various non‐motor symptoms in Parkinson’s disease [11]. A meta‐analysis also found reduced functional connectivity in the nucleus accumbens among patients with recurrent MDD [12]. Different abnormalities in neurotransmitters may represent the biological mechanisms underlying the heterogeneity of MDD. These abnormalities affect distinct brain functional activities and can be characterized through imaging methods.

Combining imaging and clustering methods has been widely used for distinguishing subtypes of MDD and investigating their mechanisms [13–16]. Tang et al. [14] utilized low‐frequency oscillation amplitude to distinguish subtypes of depression and elucidated the underlying mechanisms involving epigenetics, metabolomics, and pro‐inflammatory cytokines across different subtypes. Functional connectivity‐based subtyping revealed that MDD subtype 1 exhibited positive deviation in the parietal‐frontal cortex and default mode network (DMN) while showing negative deviations in the occipital lobe and sensorimotor network. In contrast, MDD subtype 2 demonstrated markedly opposite deviation patterns [15]. Subtype differentiation based on functional connectome deviations revealed that Subtype 1 exhibited positive deviations in the DMN, salience network, and subcortical regions while showing negative deviations in the sensorimotor network and attentional regions. Subtype 2 demonstrated moderate but opposite deviation patterns [15]. However, the above method uses unsupervised learning to cluster patients based on similarity, which makes it prone to confusing differences between individuals that are unrelated to the disease [17].

Functional gradient approaches based on connectomics are widely used to describe hierarchical classifications among brain functional networks, providing a foundation for understanding how function emerges through cortical interactions [18]. Patients with MDD exhibit global topographical alterations along primary‐to‐transmodal gradients, along with focal changes predominantly within primary and transmodal systems. These gradient alterations show significant correlations with meta‐analytic terms involving sensory processing and higher‐order cognition [19]. Following the mindfulness intervention, patients with treatment‐resistant depression exhibited reduced cortical gradient dissimilarity. Lower dissimilarity was observed between nodes in the default mode and limbic networks (LIBs), correlating with baseline levels of trait anxiety, depression, and mindfulness. Patients’ baseline LIB dissimilarity predicted trait anxiety scores at 24 weeks post‐intervention [20]. Patients with mild cognitive impairment and depressive symptoms exhibit increased functional gradients in the default‐mode network. Differences in functional connectivity gradients between patients with mild cognitive impairment and depressive symptoms and those without depressive symptoms are significantly correlated with the distribution of serotonin 5‐HT1A [21]. Compared with conventional functional connectivity analyses, which characterize pairwise interaction strengths and are susceptible to high dimensionality and noise, functional gradient mapping offers a complementary perspective by reducing the connectome to its dominant axes of variation [18]. These axes capture the macroscale hierarchical organization of the cortex—spanning from primary sensorimotor to transmodal association systems—and provide a low‐dimensional, robust representation of the brain network topology [19]. Moreover, gradient‐derived spatial patterns can be directly correlated with external molecular and cellular atlases, facilitating multimodal integration that is challenging to achieve using whole‐brain pairwise connectivity matrices [19]. Despite these advantages, and although gradient alterations have been associated with MDD symptomatology and treatment response [19], no study to date has systematically applied functional gradient features to identify neurobiologically distinct subtypes of MDD in a large, multi‐center sample.

**Figure 1:**
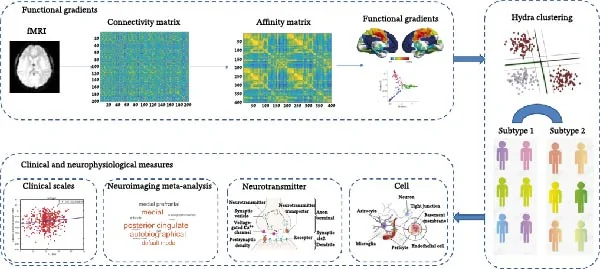
Data processing flowchart. First, pre‐process the fMRI data and calculate the functional gradients. Hydra is employed to distinguish subtypes of MDD. Subsequently, transcriptomic and clinical analyses of MDD subtypes were conducted.

We employed principal functional gradient and Hydra clustering methods to distinguish subtypes of MDD. HYDRA used a linear maximum margin classifier to classify healthy controls (HCs) and patients and employed hyperplanes to cluster patients for subtype classification. Unlike unsupervised learning, which clustered patients based on similarity and thus tended to conflate differences between individuals that are unrelated to the disease, HYDRA can effectively cluster patients based on differences between patients and controls [17]. We explored functional network gradient features and transcriptomic mechanisms across different subtypes, correlating them with clinical scales (Figure 1).

## 2. Experimental Procedures

### 2.1. Patients

The research data originate from the REST‐meta‐MDD project, a large‐scale, multi‐center imaging database for MDD [22]. Data were collected from 25 research groups across 17 Chinese hospitals. All participants agreed to provide diagnoses, age, gender, and education level. At baseline data collection, participants also provided information on first‐episode or recurrent MDD, medication status, disease duration, and 17‐item HAMD scores. All study participants provided written informed consent at their local institutions. Excluded cases included lack of information on gender, age, and education; poor imaging quality or inadequate spatial normalization as assessed by visual inspection; excessive head motion (mean frame‐to‐frame displacement >0.2 mm) or insufficient coverage (<90% of the group mask); spatial correlation <0.6 between the regional homogeneity map of each participant and the group mean ReHo map. Our study ultimately included 907 HCs and 1067 patients with MDD.

### 2.2. MRI Scanning and Data Processing

Each site in the REST‐meta‐MDD project collected high‐resolution T1‐weighted structural images and resting‐state functional magnetic resonance (rsfMRI) imaging data. The preprocessing of functional data follows a standardized analytical workflow, with specific steps detailed in the reference materials [20]. We used the Craddock 200 (CC200) brain functional atlas to extract the average time series for each subject’s region of interest. This map was generated through a data‐driven approach that spatially partitioned the entire brain into 200 functionally homogeneous regions with similar activity patterns [23]. Since CC200 does not provide brain network segmentation, we mapped CC200’s ROIs to the brain network with the largest overlapping area by calculating the size of the overlapping regions between CC200’s ROIs and Yeo’s seven functional networks (visual network [VIS], somatosensory network [SMT], dorsal attention network [DAN], ventral attention network [VAN], [LIB], frontoparietal network [FTP], and DMN) [24].

### 2.3. Functional Gradients Calculation

For the extracted CC200 functional connectivity matrix, the open‐source toolbox BrainSpace was employed to compute functional gradients [25]. The matrix underwent z‐transformation and thresholding, retaining only the top 10% weighted connections per row. A cosine similarity matrix was computed, and diffusion embedding was applied to identify the principal gradient components explaining connectivity variance in a descending order. To preserve the overall relationships between data points in the embedding space, parameter *α* was set to 0.5. Procrustes rotation was performed to align each subject’s components with the averaged template. We utilized principal functional gradients for subsequent analyses. We employed the ComBat toolbox to correct for site effects in principal functional gradients while regressing for age, education level, medication status, and head motion parameters [26].

This principal gradient was selected for subsequent subtyping analysis because (a) it explains the largest proportion of total connectome variance, offering the most parsimonious summary of whole‐brain functional organization; (b) it recapitulates a highly reproducible organizational principle across datasets, species, and analytical approaches; and (c) it has been shown to exhibit higher reliability and reproducibility than higher‐order gradients [27]. In our sample, the first three gradients explained ~47%, 26%, and 16% of the total variance, respectively.

### 2.4. Hydra Clustering

Hydra is employed to distinguish the subtypes of MDD. Hydra’s distinctive feature lies in clustering disease effects by modeling differences between patients and HCs rather than directly clustering patients. The clustering process simultaneously accounts for factors that contribute to disease heterogeneity, such as age, gender, scanner, and head motion [17]. The number of clusters was varied from 2 to 10 to determine the optimal subtype partitioning. The Adjusted Rand Index (ARI) is used to evaluate the consistency of various clustering schemes. The optimal number of clusters was determined based on the criterion that ARI peaked at the selected K and remained stable across repeated HYDRA runs, consistent with the standard model selection approach in HYDRA‐based subtyping studies [17]. To further evaluate the robustness of the identified subtypes, bootstrap resampling was conducted with 100 iterations, each sampling 80% of the participants without replacement; ARI was calculated between each bootstrap solution and the original full‐sample clustering.

### 2.5. Statistical Analysis

Statistical analysis was performed using MATLAB 2019b. We used the Mann–Whitney *U*‐test to compare whether there were significant differences in age, educational attainment, and clinical scale scores among the subjects. The Chi‐square distribution was used to compare whether there were significant differences in gender.

We used the Mann–Whitney *U*‐test to compare differences in functional gradients within the subject region and across the Yeo 7 brain networks, respectively. Multiple comparisons were corrected using the false discovery rate (FDR) adjustment with a *p*‐value threshold of 0.05. Spearman correlation analysis was used to compare the correlation between functional gradient differences and clinical indicators.

### 2.6. Neuroimaging Meta‐Analysis of Functional Gradient Differences

We uploaded the threshold Z‐maps derived from intergroup comparisons of regional functional gradient scores to neurosynth (https://neurosynth.org/) and utilized its decoder functionality to investigate associations between meta‐analytic cognitive terms and MDD‐related gradient alterations. The significance of correlation coefficients was determined via permutation tests, which corrected for spatial autocorrelation [28].

### 2.7. Correlation Analysis Between Functional Gradient Differences and Neurotransmitters

We employed the JuSpace toolbox to test whether spatial patterns of functional gradient alterations in MDD patients (relative to HC) correlate with specific neurotransmitter systems. We used z‐maps of functional connectivity gradient differences between MDD and HC groups as input, performing spatial correlation calculations with positron emission tomography (PET) and single‐photon emission computed tomography (SPECT)‐derived maps. PET and SPECT mapping within JuSpace was conducted in the MNI space. We selected radioligands targeting the 5‐HT1A, 5‐HT1B subtype (5‐HT1B), 5‐HT2A subtype (5‐HT2A), dopamine D1, dopamine D2, dopamine transporter, and norepinephrine transporter (NET). Precise *p*‐values were calculated based on 1000 permutations per analysis to test whether the observed patient‐specific correlations deviated from a zero distribution. Additionally, the spatial autocorrelation was adjusted for using the gray matter tissue probability map from Statistical Parametric Mapping (SPM12). *p*‐Values underwent FDR correction based on the number of tests per group comparison [29].

### 2.8. Correlation Analysis Between Functional Gradient Differences and Cell Types

We also employed the JuSpace toolbox to test whether spatial patterns of functional gradient alterations in MDD patients (relative to HC) correlate with specific cell types. We extracted regional cell type distributions associated with glia, neuro, and mitochondria. Input z‐maps of functional connectivity gradient differences between MDD and HC groups were used for spatial correlation calculations with cell‐type maps. Cell morphology mapping data were downloaded from the Allen Institute for Brain Science and processed using the abagen toolbox [29–33]. Continuous graphs were interpolated by assigning each voxel the distance‐weighted average of its 10 nearest neighbors. Statistical analyses were consistent with the neurotransmitter section [30–34].

### 2.9. Validation Analysis

To validate the reliability and reproducibility of these findings, we repeated our analysis process using the AAL90 atlas as a replacement for the Craddock 200 brain functional atlas. We recalculated functional gradients using the AAL90 atlas, performed Hydra clustering based on functional gradients, and conducted a neuroimaging meta‐analysis of functional gradient differences. We also excluded MDD patients taking medication and established a clinically matched HC group to conduct a validation analysis of MDD subtypes.

## 3. Results

### 3.1. Neuropsychological Results

Table 1 contains information on demographics and medical issues. The only factors that did not significantly differ between groups were age (Mann–Whitney *U* test: *z* = −1.341, *p* = 0.178), education (*z* = 0.368, *p* = 0.710), gender distribution (chi‐square test: Chi^2^ = 2, *p* = 0.157).

**Table 1:** Demographics and neuropsychological data.

Characteristic	MDD	HC	*p*‐Value	Subtype 1	Subtype 2	*p*‐Value
Gender, *n* (M/F)	1067 (385/682)	907 (375/53)	0.157	595 (203/392)	472 (182/290)	0.157
Age, years, mean (SD)	36.6 (14.3)	37.0 (14.0)	0.178	34.5 (13.8)	36.7 (14.9)	0.016
Education, years, mean (SD)	12.3 (3.1)	11.4 (4.7)	0.710	12.3 (3.1)	12.3 (3.1)	0.902
First episode/recurrence	450 (235)	—	—	263 (141)	187 (94)	—
Medicated/unmedicated	326 (394)	—	—	178 (223)	138 (171)	—
HAMD‐17	18.5 (9.2)	—	—	18.5 (9.1)	18.7 (9.3)	0.352

*Note:* Data are represented as means (standard deviations). The chi‐squared test was used to compare sex, and Mann–Whitney *U* test were used to compare age and neuropsychological data.

Abbreviations: F, female; HAMD‐17, Hamilton Depression Scale‐17; HCs, healthy controls; M, male; MDD, major depressive disorder.

### 3.2. Hydra Identified Two Subtypes of MDD

**Figure 2:**
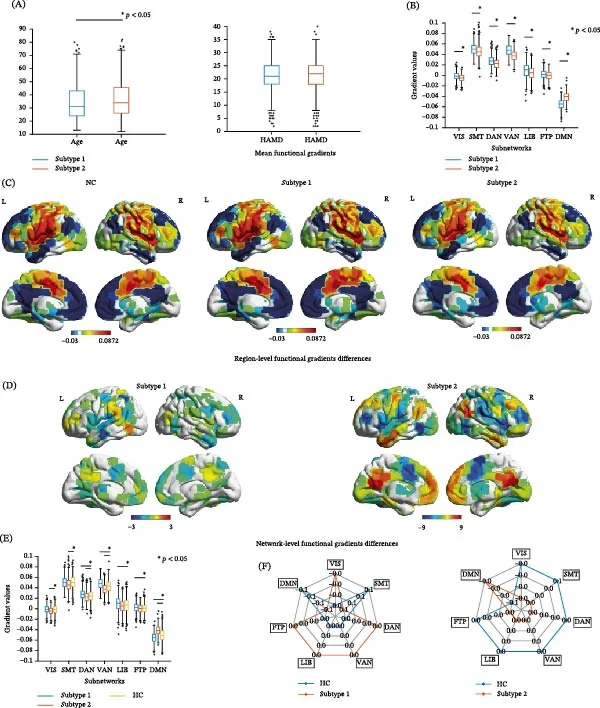
Demographic and functional gradient analysis of MDD subtypes. (A) Based on the functional gradient values of the MDD and HC groups, the Hydra clustering algorithm identified two subtypes of MDD with an ARI score of 0.3008. Subtype 1 comprised 595 MDD patients, while subtype 2 comprised 472 MDD patients. Compared to subtype 2, subtype 1 exhibited a significantly lower age distribution (*p* = 0.0119, *t* = −3.9560). There were no significant differences in HAMD parameters between the two subgroups. (B) The mean functional gradients of seven brain networks differed significantly between the two subtypes. Compared to subtype 2, subtype 1 exhibited significantly higher mean gradients in the VIS, SMT, DAN, VAN, LIB, and FTP and significantly lower mean gradients in the DMN. (C) The mean functional gradients for the MDD subtypes all exhibited a primary‐to‐transmodal brain region gradient distribution. (D) Compared with the healthy control group’s regional functional gradient, both subtypes exhibited widespread abnormalities in functional gradient. (E) Compared with the healthy control group’s network functional gradients, Subtype 1 exhibited abnormal functional gradient values in the dorsal attention network, ventral attention network, limbic network, frontoparietal network, and default mode network; Subtype 2 exhibited abnormal functional gradient values in the visual network, somatosensory network, dorsal attention network, ventral attention network, and default mode network. (F) The radar diagram corresponding to (E) illustrates the functional gradient network differences between the two subtypes and the healthy control group. DAN, dorsal attention network; LIM, limbic; SD, standard deviation; SMT, somatomotor; TPN, temporoparietal network; VAN, ventral attention network; VIS, visual network.

Based on the functional gradient values of the MDD and HC groups, the Hydra clustering algorithm identified two subtypes of MDD with a maximum ARI score of 0.3008. The stability of the two‐subtype solution was further evaluated through additional validation approaches. Bootstrap resampling (100 iterations, 80% sampling) yielded a mean ARI of 0.32 between bootstrap solutions and the original clustering, indicating moderate but consistent reproducibility. Subtype 1 comprised 595 MDD patients, while subtype 2 comprised 472 MDD patients. Compared to subtype 2, subtype 1 exhibited a significantly lower age distribution (*p* = 0.0119, *t* = −3.9560) (Table 1, Figure 2A). There were no significant differences in the HAMD parameters between the two subgroups. The mean functional gradient values of seven brain networks differed significantly between the two subtypes. Compared to subtype 2, subtype 1 exhibited significantly higher mean gradients in the VIS, SMT, DAN, VAN, LIB, and FTP and significantly lower mean gradients in the DMN (Figure 2B).

### 3.3. Abnormal Functional Gradients in the Subtypes of MDD

The mean functional gradients for the MDD subtypes all exhibited a primary‐to‐transmodal brain region gradient distribution (Figure 2C). Compared with the HC group’s regional functional gradient, both subtypes exhibited widespread abnormalities in the functional gradient (Figure 2D). Compared with the HC group’s network functional gradients, Subtype 1 exhibited abnormal functional gradient values in the DAN, VAN, LIB, FTP, and DMN; Subtype 2 exhibited abnormal functional gradient values in the VIS, SMT, DAN, VAN, and DMN (Figure 2E,F).

### 3.4. Neuroimaging Meta‐Analysis of Functional Gradient Differences

**Figure 3:**
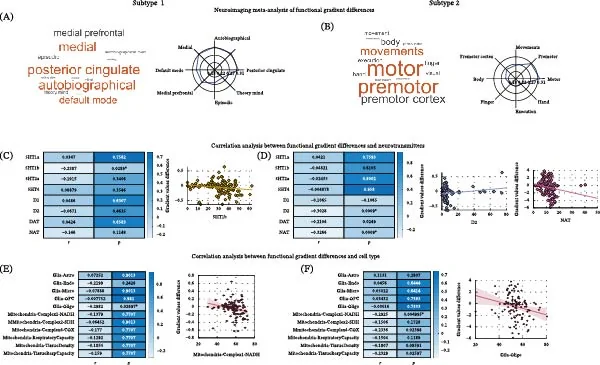
Transcriptomic analysis of MDD subtypes. (A) Abnormal functional gradients in subtype 1 correlate with cognitive and mental functions. (B) Abnormal functional gradient in subtype 2 correlate with motor and perceptual functions. (C) Spatial correlation analysis revealed that functional connectivity gradient differences in subtype 1 were significantly spatial correspondence with the normative 5‐HT1b distribution map (*p* = 0.0289, *r* = −0.2387), (D) while those in subtype 2 were significantly spatial correspondence with the normative dopamine D2 distribution map (*p* = 0.0009, *r* = −0.3028) and normative norepinephrine transporter distribution map (*p* = 0.0009, *r* = −0.3266). (E) Spatial correlation analysis with cell types revealed that functional connectivity gradient differences in subtype 1 were significantly co‐localized with the normative distribution of mitochondria‐Complex1‐NADH (*p* = 0.0049, *r* = −0.2925, uncorrected), (F) while those in subtype 2 were significantly co‐localized with the normative distribution of Glia‐Oligo (*p* = 0.0269, *r* = −0.2982).

Abnormal functional gradient values in subtype 1 correlate with cognitive and mental functions (Figure 3A). Abnormal functional gradient values in subtype 2 correlates with motor and perceptual functions (Figure 3B).

### 3.5. Correlation Analysis Between Functional Gradient Differences and Neurotransmitters

Spatial correlation analysis revealed that functional connectivity gradient differences in subtype 1 were significantly spatial correspondence with the normative 5‐HT1b distribution map (*p* = 0.0289, *r* = −0.2387) (Figure 3C), while those in subtype 2 were significantly spatial correspondence with the normative dopamine (DA) D2 distribution map (*p* = 0.0009, *r* = −0.3028) and normative NET distribution map (*p* = 0.0009, *r* = −0.3266) (Figure 3D).

### 3.6. Correlation Analysis Between Functional Gradient Differences and Cell Types

Spatial correlation analysis with cell types revealed that functional connectivity gradient differences in subtype 1 were significantly co‐localized with the normative distribution of mitochondria‐Complex1‐NADH (*p* = 0.0049, *r* = −0.2925, uncorrected) (Figure 3E), while those in subtype 2 were significantly co‐localized with the normative distribution of Glia‐Oligo (*p* = 0.0269, *r* = −0.2982) (Figure 3F).

### 3.7. Correlation Analysis Between Functional Gradient Differences and Clinical Scales

**Figure 4:**
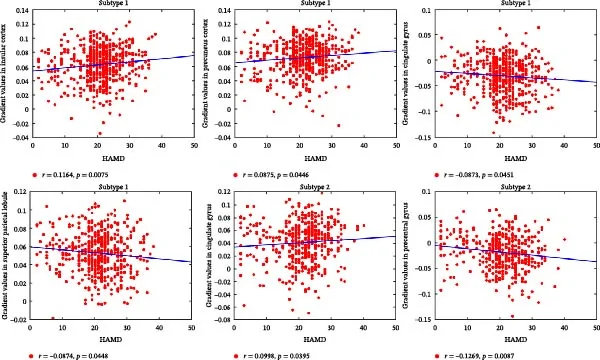
Correlation analysis between functional gradient differences and clinical scales. Subtype 1 showed a significant positive correlation with functional gradient values in the insular cortex and precuneus cortex and HAMD scores, while exhibiting a significant negative correlation with functional gradient values in the cingulate gyrus and superior parietal lobule and HAMD scores. Subtype 2 showed a significant positive correlation with functional gradient values in the cingulate gyrus and HAMD scores, while exhibiting a significant negative correlation with functional gradient values in the precentral gyrus and HAMD scores.

Subtype 1 showed a significant positive correlation with functional gradient values in the insular cortex and precuneus and HAMD scores while exhibiting a significant negative correlation with functional gradient values in the cingulate gyrus and superior parietal lobule and HAMD scores. Subtype 2 showed a significant positive correlation with functional gradient values in the cingulate gyrus and HAMD scores while exhibiting a significant negative correlation with functional gradient values in the precentral gyrus and HAMD scores (Figure 4).

### 3.8. Results of the Validation Analysis

By utilizing the AAL90 atlas, we obtained results highly similar to those from the CC200 atlas. Based on the functional gradient values of the MDD and HC groups, the Hydra clustering algorithm identified two subtypes of MDD. Compared to subtype 2, subtype 1 exhibited a significantly lower age distribution (*p*  < 0.0001, *t* = 3.5558) (Supporting Information: Figure S1A). There were no significant differences in HAMD parameters between the two subgroups (Supporting Information: Figure S1B). The mean functional gradients for the MDD subtypes all exhibited a primary‐to‐transmodal brain region gradient distribution (Supporting Information: Figure S1C). Compared with the HC group’s regional functional gradient, both subtypes exhibited widespread abnormalities in the functional gradient (Supporting Information: Figure S1D). Compared with the HC group’s network functional gradients, Subtype 1 exhibited abnormal functional gradient values in the DAN, VAN, LIB, FTP, and DMN; Subtype 2 exhibited abnormal functional gradient values in the VIS, SMT, DAN, VAN, and DMN (Supporting Information: Figure S1D). Abnormal functional gradient values in subtype 1 correlate with cognitive and mental functions. Abnormal functional gradient values in subtype 2 correlate with motor and perceptual functions (Supporting Information: Figure S1E). The results of the subtype analysis for MDD patients who had not taken medication were consistent with those of the subtype analysis that included all MDD patients (Supporting Information: Table S1 and Figure S2).

## 4. Discussion

We utilized functional gradient analysis to classify MDD subtypes, with the Hydra method revealing the two most optimized subtypes of MDD. Statistical and transcriptomic analyses indicate that subtype 1 is associated with cognitive and comprehension functions, while subtype 2 is associated to sensory and motor functions.

Cognitive impairment is a common residual feature of depression and may persist during remission. Cognitive deficits may also hinder functional recovery in individuals with MDD, including work performance [35]. Patients with MDD exhibit impaired executive function and deficits in theory of mind, specifically the ability to infer others’ mental states [36]. Impairments in the theory of mind in MDD are evident across responses to various theory of mind tasks, and theory of mind deficits show a significant correlation with the severity of depressive symptoms.

Patients with MDD exhibit abnormalities in visual cortex function, and the effects of several antidepressants correlate with improvements in visual cortex structure and synaptic function [37]. Following antidepressant treatment, left premotor cortex thickness decreased in patients with MDD and showed a significant correlation with improvement in HAMD scores [38]. Compared with healthy volunteers, patients with MDD exhibited reduced global brain connectivity in sensorimotor/visual networks and increased global brain connectivity primarily within the DMN. These key findings were consistent across different clinical states of MDD [39].

The 5‐HT1B receptor belongs to the class of G‐protein‐coupled receptors and is primarily localized at axon terminals [40]. In rat models of hereditary depressive traits (Flinder sensitive line) and rats separated from their mothers, 5‐HT1B receptor binding rates in the hippocampus are reduced. The influence of genetic or environmental susceptibility to depression on 5‐HT1B receptor binding can be reversed by antidepressants [41]. In the HC group, a statistically significant positive correlation was observed between creative ability and the average availability of 5‐HT1B receptors in the gray matter [42].

Research using SPECT has demonstrated that patients with MDD exhibit increased binding at striatal D2 receptors compared to control subjects [43]. Following duloxetine intervention, a significant positive correlation was observed between changes in NET availability in MDD patients and HAMD scores. Patients with greater reductions in HAMD scores exhibited more pronounced decreases in NET availability [44]. Dopamine primarily influences motor function and performance through dopamine receptors (D1 and D2). Abnormal beta‐band oscillations have been detected in the primary motor cortex of Parkinson’s patients, and these are associated with dopamine depletion [45].

Microglia are the primary immune cells of the central nervous system and play a crucial role in normal brain function. They exhibit functional diversity, regulating inflammation within the central nervous system by releasing inflammatory cytokines, phagocytosing and clearing apoptotic cells, and modulating synaptic plasticity and neural network formation through synaptic pruning. The dysfunction of microglia is associated with various neuropsychiatric disorders [46]. Alterations in extracellular DA targeting DA receptors affect not only neurons but also oligodendrocytes (OLs). During myelination, the two DA receptors, D2 and D3, are primarily expressed in mature OLs and OL precursor cells, playing a crucial role in myelin maintenance [47]. Within the central nervous system, OL precursor cells primarily differentiate into OLs, producing myelin sheaths that envelop portions of axons. Chronic mild stress mouse models exhibit significantly reduced myelin and OL‐associated proteins, alongside stress‐induced depressive‐like behaviors and white matter defects [48].

It is critical to note that the absence of significant differences in total HAMD scores and the lack of treatment follow‐up data preclude direct clinical application of these subtypes [49]. Our findings should be interpreted as a pathophysiological heuristic rather than a ready‐to‐use clinical decision tool [50]. The significant correlations with distinct neurotransmitters (5‐HT1B vs. DRD2/NET) and cell types (mitochondria vs. OLs) generate strong hypotheses that specific pharmacological agents (e.g., 5‐HT1B modulators vs. DRD2/NET‐targeting drugs) might yield differential efficacy across subtypes, which await verification in future randomized controlled trials.

It is also important to recognize that MDD heterogeneity arises from complex interactions among genetic susceptibility, environmental exposures, and neurobiological alterations rather than from neural circuit disruptions alone. Recent studies have emphasized that integrating environmental exposures—ranging from childhood adversity to psychosocial stressors—with neuroimaging phenotypes is essential for understanding the full spectrum of depression heterogeneity [51]. Likewise, genetically informed approaches have provided critical insights into the biological underpinnings of psychiatric heterogeneity and treatment response variability [52]. Emerging evidence further demonstrates that multimodal frameworks combining genetic, environmental, and neuroimaging data can yield more robust and clinically meaningful MDD subtypes than any single modality alone [53]. In this context, our current findings—derived solely from resting‐state functional MRI—should be interpreted as one dimension of MDD heterogeneity rather than comprehensive biological subtypes. The gradient‐based stratification we propose provides a neurobiologically grounded heuristic that prioritizes specific neural circuits and neurotransmitter systems for further investigation. However, we acknowledge that integrating genetic risk scores, environmental exposure histories, detailed symptom dimensions, and longitudinal treatment outcomes will be essential for developing more complete and clinically actionable subtyping frameworks in future studies.

Our study has several limitations. First, templates based on neurotransmitters and cell types derived from HCs may not explain the neurotransmitter and cell type characteristics observed in patients with MDD. Second, the study revealed brain abnormalities associated with cognitive function; however, the data did not include cognitive function scales, making it impossible to further evaluate outcomes related to cognitive function. Third, the data currently in use are cross‐sectional large‐sample data, requiring longitudinal studies to explore the longitudinal developmental trajectories of MDD subtypes. Finally, while the JuSpace analyses provide valuable biological contextualization, the reliance on normative PET/SPECT and post‐mortem histology templates limits the ability to draw direct causal inferences about neurotransmitter or cellular dysfunction. The observed spatial overlaps are associative in nature and may be influenced by the inherent spatial autocorrelation of both imaging and molecular data. Direct validation via concurrent PET‐fMRI or SPECT in patient cohorts is necessary to substantiate these subtype‐specific molecular hypotheses.

## 5. Conclusions

We identified two subtypes of MDD and characterized their functional gradient features. Transcriptomic studies revealed the cognitive terminology meta‐analysis, neurotransmitter, and cell type characteristics of these two subtypes.

## Author Contributions

Xiaozheng Liu analyzed the data, wrote and revised this manuscript. Zhongwei Guo designed the experiments.

## Funding

This research was supported by the General Project of the Department of Science and Technology of Zhejiang Province (Grant 2020KY182 to Xiaozheng Liu) and the General Project of the Department of Science and Technology of Zhejiang Province (Grant 2018KY031 and Grant 2024KY873 to Zhongwei Guo).

## Disclosure

All authors have participated in this research and approved the final manuscript.

## Ethics Statement

All participants in all datasets provided written informed consent. All recruitment procedures and experimental protocols were approved by the institutional review boards of the principal investigators’ respective institutions. This study was conducted in accordance with the Declaration of Helsinki.

## Consent

The authors have nothing to report.

## Conflicts of Interest

The authors declare no conflicts of interest.

## Supporting Information

Additional supporting information can be found online in the Supporting Information section.

## Supporting information


**Supporting Information** This section collects Supporting Information included in this article. Table S1: Demographics and neuropsychological data. Figure S1: Validation analysis results of MDD subtype identification based on the AAL90 atlas. (A) Compared to subtype 2, subtype 1 exhibited a significantly lower age distribution (*p* < 0.0001, *t* = 3.5558). (B) There were no significant differences in HAMD parameters between the two subgroups. (C) The mean functional gradients for the MDD subtypes all exhibited a primary‐to‐transmodal brain region gradient distribution. Compared with the healthy control group’s regional functional gradient, both subtypes exhibited widespread abnormalities in functional gradient. (D) Compared with the healthy control group’s network functional gradients, Subtype 1 exhibited abnormal functional gradient values in the dorsal attention network, ventral attention network, limbic network, frontoparietal network, and default mode network; Subtype 2 exhibited abnormal functional gradient values in the visual network, somatosensory network, dorsal attention network, ventral attention network, and default mode network. (E) Abnormal functional gradient values in subtype 1 correlate with cognitive and mental functions. Abnormal functional gradient values in subtype 2 correlate with motor and perceptual functions. Figure S2: Validation analysis results of MDD subtype identification based on MDD patients not taking medication. (A) Compared to subtype 2, subtype 1 exhibited a significantly lower age distribution (*p* = 0.0484, *t* = 1.9740). (B) There were no significant differences in HAMD parameters between the two subgroups. (C) The mean functional gradients for the MDD subtypes all exhibited a primary‐to‐transmodal brain region gradient distribution. (D) Compared with the healthy control group’s regional functional gradient, both subtypes exhibited widespread abnormalities in functional gradient. Compared with the healthy control group’s network functional gradients, Subtype 1 exhibited abnormal functional gradient values in the dorsal attention network, ventral attention network, limbic network, frontoparietal network, and default mode network; Subtype 2 exhibited abnormal functional gradient values in the visual network, somatosensory network, dorsal attention network, ventral attention network, and default mode network. (E) Abnormal functional gradient values in subtype 1 correlate with cognitive and mental functions. Abnormal functional gradient values in subtype 2 correlate with motor and perceptual functions.

## Data Availability

Data of the REST‐meta‐MDD Project are available at: https://rfmri.org/REST-meta-MDD. Software packages used in this manuscript include Hydra (https://github.com/evarol/HYDRA), Functional gradients (https://github.com/MICA-MNI/BrainSpace), and ComBat harmonization (https://github.com/Jfortin1/ComBatHarmonization).
