# Supplementary material for: Connectome Gradient‐Based Subtyping of Major Depressive Disorder Reveals Distinct Neurobiological and Transcriptomic Signatures
Source: Depress Anxiety. 2026 Sep 2;2026:8792696. doi: 10.1155/da/8792696 (PMC13536004; doi:10.1155/da/8792696)
Supplement: Supplementary file 1 [file DA-2026-8792696-s001.docx]

**Statistical analysis**

MATLAB 2019b was used to perform statistical comparisons of clinical data between the group of patients with MDD who were not taking medication and the healthy control group. We used the Mann-Whitney U-test to compare whether there were significant differences in age, educational attainment, and clinical scale scores among the subjects. The chi-square distribution was used to compare whether there were significant differences in gender.

We used the Mann-Whitney U-test to compare differences in functional gradients within the subject region. Multiple comparisons were corrected using false discovery rate (FDR) adjustment with a p-value threshold of 0.05.

**Neuroimaging meta-Analysis of functional gradient differences**

We uploaded the threshold Z-maps derived from intergroup comparisons of regional functional gradient scores to neurosynth (https://neurosynth.org/) and utilized its decoder functionality to investigate associations between meta-analytic cognitive terms and MDD-related gradient alterations. The significance of correlation coefficients was determined via permutation tests, which corrected for spatial autocorrelation [28].

**Neuropsychological results**

Table S1 contains information on demographics. The factors that did not significantly differ between groups were age (Mann-Whitney U test: z = -0.645, p = 0.519), education (z = 0.279, p = 0.780), gender distribution (chi-square test: c2 = 1.050, p = 0.305).

**Results of the validation analysis**

By utilizing the AAL90 atlas, we obtained results highly similar to those from the CC200 atlas. Based on the functional gradient values of the MDD and HC groups, the Hydra clustering algorithm identified two subtypes of MDD. Compared to subtype 2, subtype 1 exhibited a significantly lower age distribution (p < 0.0001, t = 3.5558) (Figure S1A). There were no significant differences in HAMD parameters between the two subgroups (Figure S1B). The mean functional gradients for the MDD subtypes all exhibited a primary-to-transmodal brain region gradient distribution (Figure S1C). Compared with the healthy control group's regional functional gradient, both subtypes exhibited widespread abnormalities in functional gradient (Figure S1D). Compared with the healthy control group's network functional gradients, Subtype 1 exhibited abnormal functional gradient values in the dorsal attention network, ventral attention network, limbic network, frontoparietal network, and default mode network; Subtype 2 exhibited abnormal functional gradient values in the visual network, somatosensory network, dorsal attention network, ventral attention network, and default mode network (Figure S1D). Abnormal functional gradient values in subtype 1 correlate with cognitive and mental functions. Abnormal functional gradient values in subtype 2 correlate with motor and perceptual functions (Figure S1E). The results of the subtype analysis for MDD patients who had not taken medication were consistent with those of the subtype analysis that included all MDD patients (Supplementary Materials: Table S1 and Figure S2).

**Table S1.** Demographics and neuropsychological data

|  | MDD | HC | *P value* | Subtype1 | Subtype2 | *P value* |
| --- | --- | --- | --- | --- | --- | --- |
| Gender, n (M/F) | 741(276/465) | 752(310/441) | 0.305 | 329(136/193) | 412(140/272) | 0.349 |
| Age, years, mean(SD) | 32.1(11.8) | 31.6(11.3) | 0.519 | 31.1(11.3)_ | 33(12.1) | 0.380 |
| Education, years, mean(SD) | 12.5(3.0) | 11.5(4.8) | 0.780 | 12.6(3.1) | 12.5(2.9) | 0.902 |
| First episode/Recurrence | 364(137) | - | - | 234(132) | 166(85) | 0.598 |
| HAMD-17 | 21.8(6.3) | - | - | 22.0(6.4) | 21.7(6.2) | 0.709 |

Data are represented as means (standard deviations). The chi-squared test was used to compare sex, and Mann-Whitney U test were used to compare age and neuropsychological data. *HCs*, healthy controls; *MDD*, Major depressive disorder; *M*, Male; *F*, Female; *HAMD-17*, Hamilton Depression Scale-17.

**Figure S1.** Validation analysis results of MDD subtype identification based on the AAL90 atlas. **(A)** Compared to subtype 2, subtype 1 exhibited a significantly lower age distribution (p < 0.0001, t = 3.5558). (B) There were no significant differences in HAMD parameters between the two subgroups. (C) The mean functional gradients for the MDD subtypes all exhibited a primary-to-transmodal brain region gradient distribution. Compared with the healthy control group's regional functional gradient, both subtypes exhibited widespread abnormalities in functional gradient. (D) Compared with the healthy control group's network functional gradients, Subtype 1 exhibited abnormal functional gradient values in the dorsal attention network, ventral attention network, limbic network, frontoparietal network, and default mode network; Subtype 2 exhibited abnormal functional gradient values in the visual network, somatosensory network, dorsal attention network, ventral attention network, and default mode network. (E) Abnormal functional gradient values in subtype 1 correlate with cognitive and mental functions. Abnormal functional gradient values in subtype 2 correlate with motor and perceptual functions.

**Figure S2.** Validation analysis results of MDD subtype identification based on MDD patients not taking medication. **(A)** Compared to subtype 2, subtype 1 exhibited a significantly lower age distribution (p = 0.0484, t = 1.9740). (B) There were no significant differences in HAMD parameters between the two subgroups. (C) The mean functional gradients for the MDD subtypes all exhibited a primary-to-transmodal brain region gradient distribution. (D) Compared with the healthy control group's regional functional gradient, both subtypes exhibited widespread abnormalities in functional gradient. Compared with the healthy control group's network functional gradients, Subtype 1 exhibited abnormal functional gradient values in the dorsal attention network, ventral attention network, limbic network, frontoparietal network, and default mode network; Subtype 2 exhibited abnormal functional gradient values in the visual network, somatosensory network, dorsal attention network, ventral attention network, and default mode network. (E) Abnormal functional gradient values in subtype 1 correlate with cognitive and mental functions. Abnormal functional gradient values in subtype 2 correlate with motor and perceptual functions.
